# Mycobiome of Stool, Blood, Thrombus and Vessel Wall in Abdominal Aortic Aneurysm Patients

**DOI:** 10.1007/s11046-025-01002-z

**Published:** 2025-10-03

**Authors:** Éva Nemes-Nikodém, Gergő Péter Gyurok, Zsuzsanna A. Dunai, Nóra Makra, Bálint Hofmeister, Dóra Szabó, László Hidi, Ágnes Szappanos, Gergely Imre Kovács, Péter Sótonyi, Eszter Ostorházi

**Affiliations:** 1 Department of Medical Microbiology, Semmelweis University, Budapest, Hungary; 2 Department of Vascular and Endovascular Surgery, Heart and Vascular Center, Semmelweis University, Budapest, Hungary; 3 HUN-REN-SU Human Microbiota Research Group, Budapest, Hungary; 4 Department of Rheumatology and Clinical Immunology, Semmelweis University, Budapest, Hungary; 5 Department of Dermatology, Venereology and Dermatooncology, Semmelweis University, Budapest, Hungary

**Keywords:** Mycobiome, Abdominal aortic aneurysm, ITS, Gut-blood-vessel wall axis, *Tomentella*, *Malassezia*

## Abstract

Abdominal aortic aneurysm (AAA) is a life-threatening vascular condition characterized by inflammatory degeneration of the vessel wall. Emerging evidence suggests that microbial factors contribute to its progression. In this study, we analyzed the mycobiome composition of stool, blood, thrombus and damaged vessel wall samples collected during surgery from 24 AAA patients using Internal Transcribed Spacer (ITS) sequencing. Significant differences in alpha and beta diversity were observed across the sample types, confirming compartmentalization of the mycobiome. However, individual fungal profiles did not establish a clear gut-blood-vessel wall axis, indicating that fungi translocated to the vessel wall may originate from other anatomical regions. Comparison AAA mycobiome with healthy arterial walls’ mycobiome from organ donors revealed a dominance of anti-inflammatory *Tomentella* in healthy samples, while pro-inflammatory *Malassezia* species were prevalent in damaged vessel walls. These findings highlight the role of fungi in AAA progression and suggest potential thera-peutic avenues, including antifungal adjuvant treatments to mitigate inflammation and aneurysm development.

## Introduction

Abdominal aortic aneurysm (AAA) is a pathological lesion characterized by the progressive dilation of the aorta below the level of the renal arteries, enlarging by at least 150% compared to the relatively normal diameter of the adjacent artery [1]. The pathological changes in the vascular wall are caused by inflammation mediated by immune cells and degradation of the medial layer [2]. The early asymptomatic nature of AAA often leads to its being overlooked until it is at risk of rupture. The indication for elective surgery for AAA is if it is ≥ 5 cm in women and ≥ 5.5 cm in men [3]. The mortality rate of ruptured AAA exceeds 80% [4]. The main risk factors for the development of AAA are smoking, male gender, advanced age, hypertension, hypercholesterolemia, obesity and various cardiovascular/peripheral diseases [5]. These characteristics are common in the population, and only the knowledge of family history can highlight the importance of screening tests in a given person. Several studies have demonstrated that the bacterial composition of the gut microbiome plays a role in the progression of risk factors such as hypertension, metabolic diseases, and cardiovascular diseases [6–8]. It has also been proven that the transplantation of dysbiotic human feces from AAA patients increases, while the transplantation of healthy microbiota slows the development of AAA in mice [9]. Microbiota can directly or indirectly influence inflammatory destruction of the vascular wall. Indirectly, as circulating microbial metabolites contribute to inflammatory and apoptotic processes, or directly, as the translocated microbiota induces increased infiltration of inflammatory cells into the blood vessel [10, 11]. In our previous study, we demonstrated that the bacterial microbiome composition of the healthy vessel wall and the AAA vessel wall is different. We have shown that the different metabolic activities of bacteria characteristic of damaged or healthy vascular walls can have a direct aggravating or protective effect on aneurysm progression. We also experienced that the origin of bacterial genera with high abundance in the aneurysmal vessel wall cannot be confirmed by gut microbiome translocation via the blood [12].

Despite the fact, that gut fungi constitute less than 1% of the total microbial population, they are essential for maintaining host health and modulating disease states. The intestinal mycobiome influences nutrient absorption and metabolism through unique metabolic pathways and modulates the host immune system [13, 14]. Parallel with associations between the composition of the gut bacterial microbiome and AAA risk factor diseases, some researchers have also found a relationship between the composition of the fecal mycobiome and the aforementioned conditions [15–17].

The study of Yao et al. investigated the role of gut fungal communities in the development of AAA. Significant dysbiosis was observed in the gut mycobiomes of AAA patients compared to healthy individuals. The abundance of *Candida* species increased, and the abundance of *Saccharomyces cerevisiae* decreased in the gut mycobiome of AAA patients [18].

As a continuation of our previous research on the role of the bacterial microbiome in the development and progression of abdominal aortic aneurysm (AAA), the present study aims to investigate the composition of the fungal microbiome. In the current study, we investigated the mycobiome composition of stool, blood, thrombus, and aneurysm from 24 AAA patients. We compared mycobiome abundances from different samples from individuals to investigate whether fungi found in thrombus and AAA vessel wall could be derived from stool via the blood-mediated route. We compared the mycobiome composition of the healthy vessel wall with the AAA mycobiome composition to identify fungi that play protective or detrimental roles against pathological tissue changes.

## Materials and Methods

### Sample Collection

We prospectively enrolled 24 patients with AAA who underwent surgery at the Department of Vascular and Endovascular Surgery, Heart and Vascular Center, Semmelweis University, between March 2022 and February 2023. The study was conducted in accordance with the Declaration of Helsinki and approved by the Ethics Committee of Semmelweis University based on the Research Authorization Decisions (EIÜG) of the Hungarian National Center for Public Health (9882-8/2022 EÜIG,/23.03.2022). Written informed consent was obtained from all patients involved in the study. All study participants gave written informed consent that data from their personal test results could be published. Data and test results in the manuscript cannot be linked to the individual participants, all tests were anonymized. Inclusion criteria were age over 18 years, legal capacity, abdominal aortic aneurysm, indication for open surgery, and presence of thrombus in the aorta. Aneurysmal wall and intraluminal thrombus were carefully sampled to avoid contamination during open aortic repair surgery. During the open abdominal surgery, all applicable aseptic and antiseptic protocols are strictly followed to prevent iatrogenic infection in the patient undergoing the procedure. Approximately 3 g of intraluminal thrombus and aneurysm wall were collected into sterile, DNase and RNase free, low binding microfuge tubes (AM12450 Invitrogen Microfuge tube, Thermo Fisher Scientific, Waltham, MA, USA) to avoid potential contamination. Blood from these samples was washed with sterile saline and then frozen in liquid nitrogen. At least 3000 µL of whole blood was collected in citrate-filled VACUETTE collection tubes (Greiner Bio-One, Stonehouse, UK) and immediately frozen at − 80 °C after tube rotation. Stool sample collection were taken place after anesthesia using a perianal swab. All samples were stored at − 80 °C until Deoxyribonucleic acid (DNA) extraction. Twelve human femoral arteries previously collected from donors during multi-organ donation [19] were enrolled in mycobiome study to examine as healthy negative controls. In both the aortic aneurysm group and the healthy negative controls, taking antibiotics, antifungals, or probiotics in the previous month was an exclusion criterion from the study.

### DNA Extraction

DNA isolation was performed by NucleoSpin Blood, Mini kit (Macherey–Nagel, Allentown, PA, USA) from blood samples and by ZymoBIOMICS DNA Miniprep Kit (Zymo Research Corp., Irvine, CA, USA) from aneurysm, thrombus and stool samples according to the manufacturer’s instructions, after enzymatic dissolution with 20 µL of Proteinase K solution in 200 µL of lysis buffer (56 °C, 3 h). Concentration of genomic DNA was measured using a Qubit2.0 Fluorometer with Qubit 117 dsDNA HS Assay Kit (Thermo Fisher Scientific, Waltham, MA, USA).

### ITS (Internal Transcribed Spacer) Mycobiome Analysis

 The standard Illumina fungal metagenomic protocol was modified to perform the ITS mycobiota analysis (https://support.illumina.com/content/dam/illumina-support/documents/documentation/chemistry_documentation/metagenomic/fungal-metagenomic-demonstrated-protocol-1000000064940-01.pdf). Polymerase chain reaction (PCR) was optimized using increased amount of purified DNA of 6.25 μL per reaction and using decreased volume of primers of 3 μL to minimize primer dimer formation. The volume of 12.5 µL of 2 × KAPA HiFi HotStart ReadyMix as well as the total PCR reaction volume (25 µL) were kept unchanged. The number of PCR cycles were increased to 30 for adequate amplification of the target regions. Non-specific products and primer dimers were removed with a two-step purification using 25 μL and 10 μL beads sequentially, which supported the retention of the target amplicon with QuantaBio SparQ PureMag Beads (QIAGEN, Germantown, ML, USA). To avoid contamination and to increase the reliability of the study, all analysis procedures were done in duplicate. To evaluate the contribution of extraneous DNA from reagents, extraction negative controls and PCR negative controls were included in every run. Throughout the DNA isolation process, nuclease-free water was employed as a negative control, while the ZymoBIOMICS Microbial Community DNA Standard served as the positive control (Zymo Research Corp., Irvine, CA, USA). PCR product libraries were assessed using DNA 1000 Kit with Agilent 2100 Bioanalyzer (Agilent Technologies, Waldbronn, Germany). Equimolar concentrations of libraries were pooled and sequenced on an Illumina MiSeq platform (Illumina, San Diego, CA, USA) using MiSeq Reagent Kit v3 (600 cy-cles PE). Raw sequencing data were retrieved from the Illumina BaseSpace and the data were analysed by the CosmosID bioinformatics platform (Cosmosid Inc., Germantown, ML, USA) [20].

### Statistical Analysis

 The Wilcoxon Rank Sum test for Chao1 Alpha diversity, and PERMANOVA analysis for Jaccard Principal Coordinates Analysis (PCoA) Beta diversity was used for statistical testing between cohorts of samples, by applying the statistical analysis support of CosmosID bioinformatics platform [20]. Statistical significance was decided upon at a two-tailed *p* value of ≤ 0.05.

### Data Availability

The datasets generated and analyzed during the current study are available in the SRA repository: SRA/PRJNA1139113/www.ncbi.nlm.nih.gov (accessed on 22 October 2024).

## Results

From the extraction negative controls and PCR negative controls processed simultaneously with the samples, neither DNA isolation nor ITS PCR resulted in measurable amounts of DNA. In all vessel walls, thrombus, blood and stool samples of our 24 aortic aneurysm patients and 12 healthy vessel walls included in the study, library preparation and sequencing were successful by amplifying the ITS region. The average length of index ITS PCR products was 724 bp measured by Agilent 2100 Bioanalyzer. The number of reads varied between 125,995 minimum and 463,713 maximum values per sample. The amount of fungi identifiable at the genus level within a sample varied between 53 and 100%, with the remaining reads falling into the unidentified fungal genus group. However, we do not consider some fungal genus identifications to be valid results, as it is difficult to accept the fact that mushroom DNA was identified in blood, thrombus, and vascular wall. The results of these misidentifications were the genera *Helotiales, Leotiomycetes* and *Russula* which were not considered real results. In our further comparative studies, we also classified these presumably misidentified taxa as unidentifiable fungi.

**Fig. 1:**
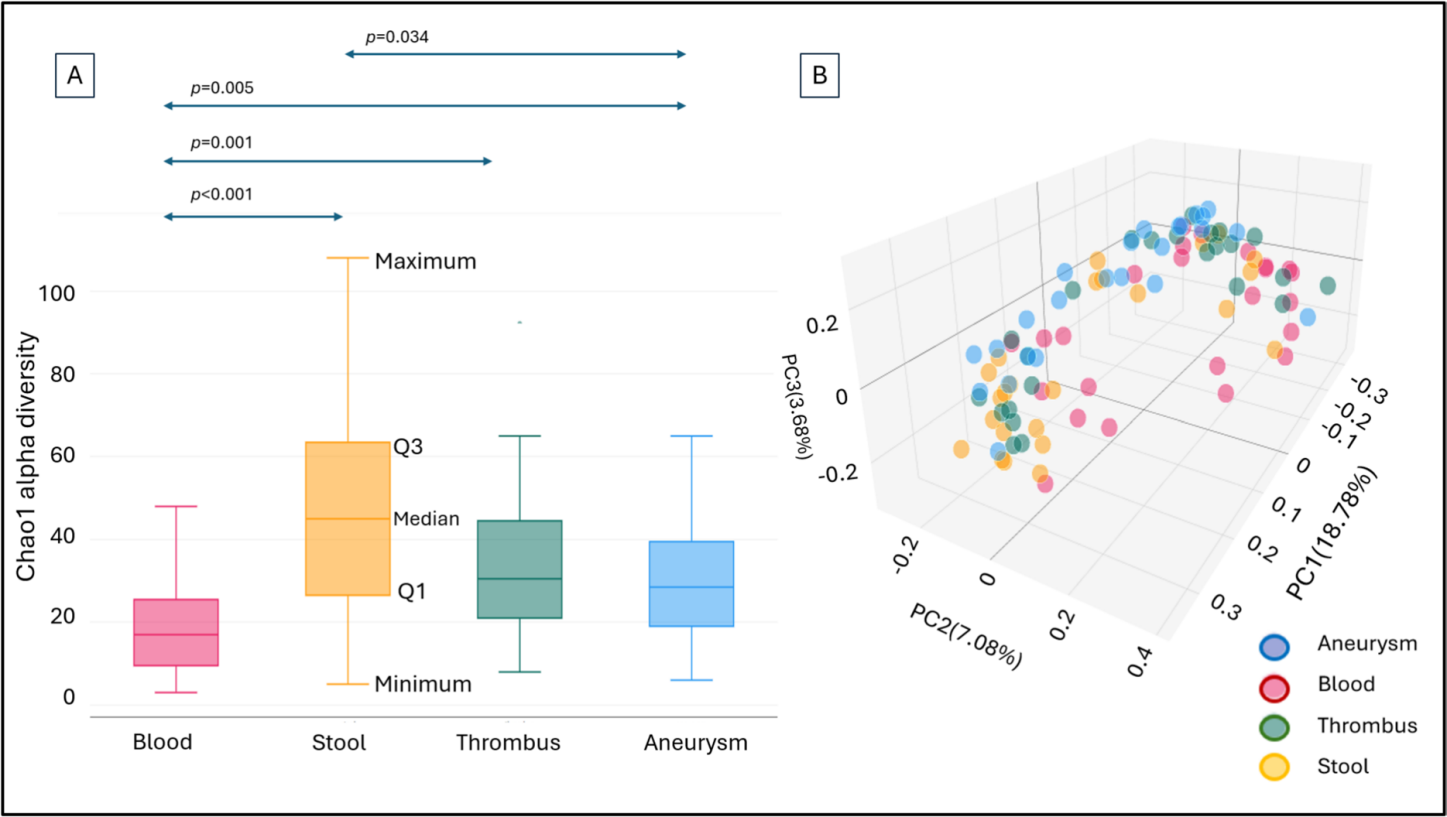
Mycobiome Chao1 alpha diversity (**A**) and Jaccard beta diversity PCoA (**B**) of blood, stool, thrombus and aneurysm wall samples of AAA patients: Boxplot displaying the data distribution with minimum, first quartile (Q1), median, third quartile (Q3), and maximum values, illustrating central tendency and variability, PC: principal coordinate

The mycobiome composition of the different sample types gave different results in both alpha and beta diversity (Fig. 1). The Chao1 alpha diversity levels were significantly different between blood and stool samples (*p* < 0.001), between blood and thrombus samples (*p* = 0.001), between blood and aneurysm samples (*p* = 0.005) and between stool and aneurysm samples (*p* = 0.034). Although the sets of sample type cohorts overlap in the Jaccard beta diversity PCoA plot, the PERMANOVA statistical calculation still demonstrates significant differences between blood-stool, blood-aneurysm, stool-thrombus and stool-aneurysm samples with the *p* value of 0.002, 0.004, 0.032 and 0.034, respectively.

**Fig. 2:**
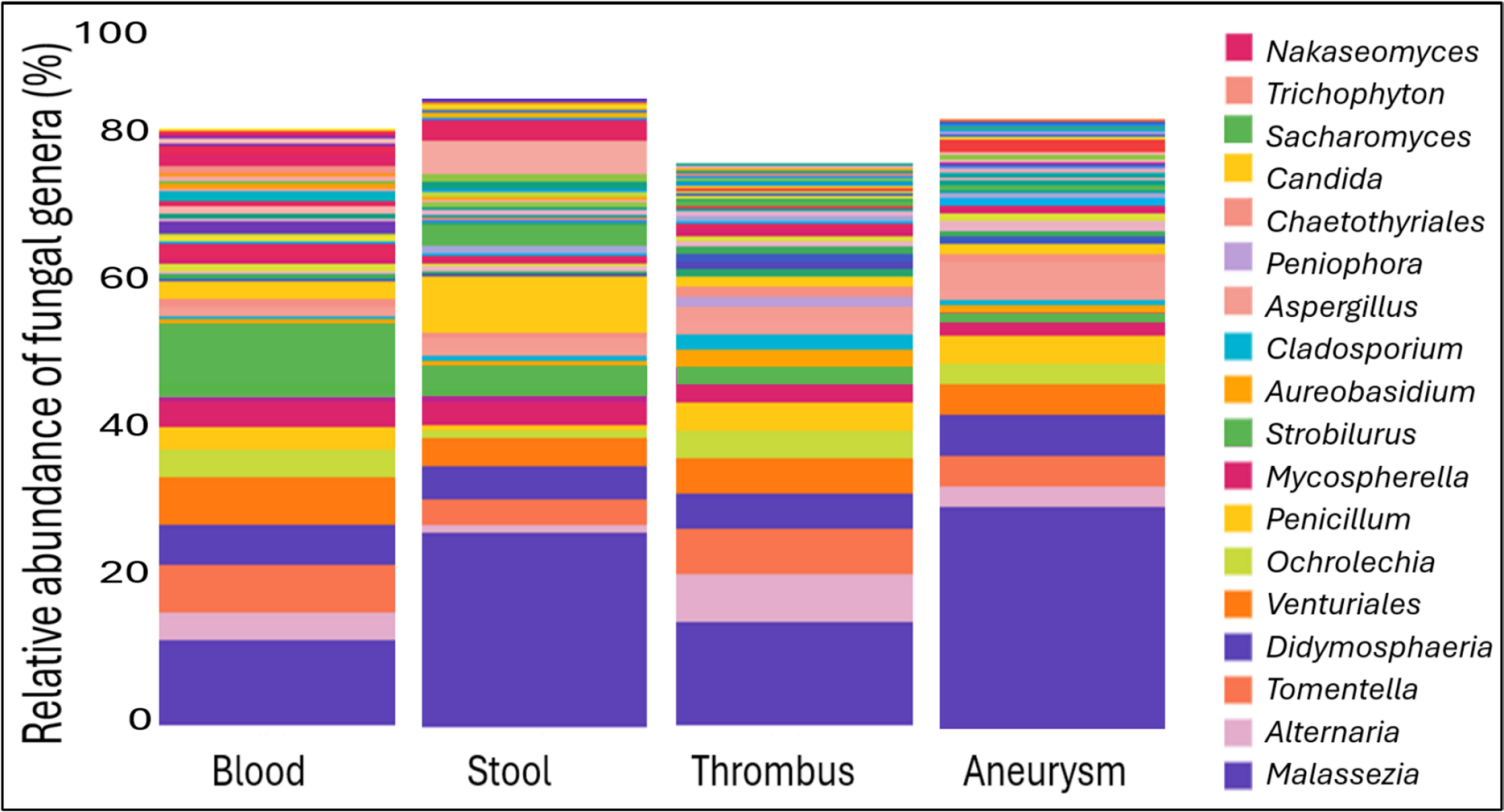
Average abundance of the most common fungal genera in the different sample types

The average fungal genus abundance ratios for the aggregated cohorts of each sample type (blood, stool, thrombus, aneurysm) differ, but due to the high standard deviation of the individual samples, the differences are not significant. Figure 2 shows the average abundance of the most common fungal genera in the different sample types.

**Fig. 3:**
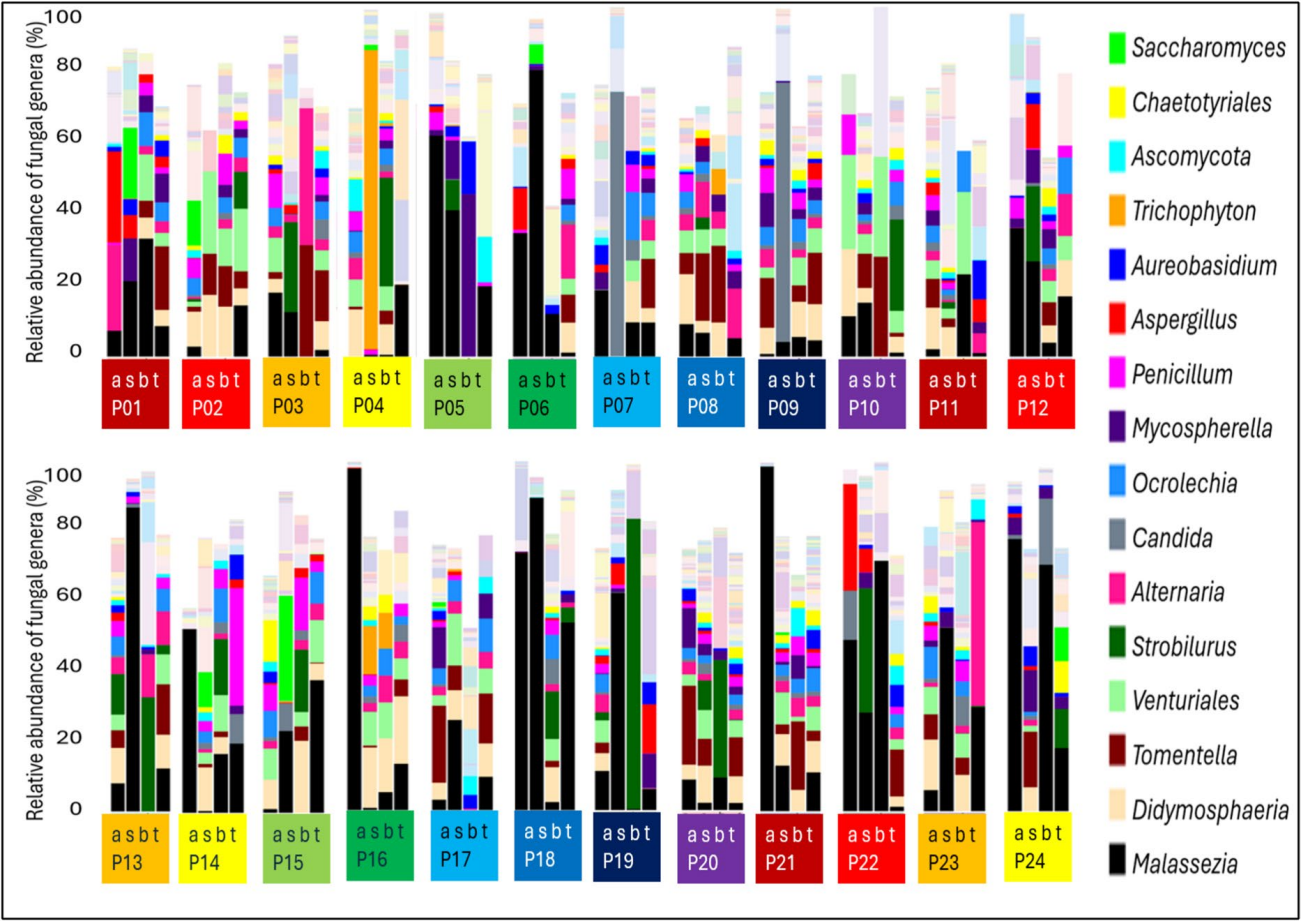
Stacked bar plot of fungal genus abundance for 4 sample types from each individual: a-aneurysm, b-blood, s-stool, t-thrombus

No fungal genus can be identified whose dominant abundance is characteristic of a given sample type. In the same way, examining the 4 sample types per person in parallel, a person-specific mycobiome composition cannot be verified. Comparing the individual fungal genus abundance of patients across the four sample types (Fig. 3), there are individuals who show a similar distribution (P08, P20) and others whose samples are very different from each other (P04, P07). In some patients with an abundance of the *Malassezia* genus above 20% in the aneurysm sample, the stool *Malassezia* abundance is also high (P5, P6, P12, P18) and this is accompanied by a high abundance of *Malassezia* in the blood (P22). Opposite examples are patients P14, P16, P21, in whom high abundance of *Malassezia* is only characteristic of the aneurysm wall among the samples, and in patient P24, high abundance of *Malassezia* is seen in the aneurysm and blood.

**Fig. 4:**
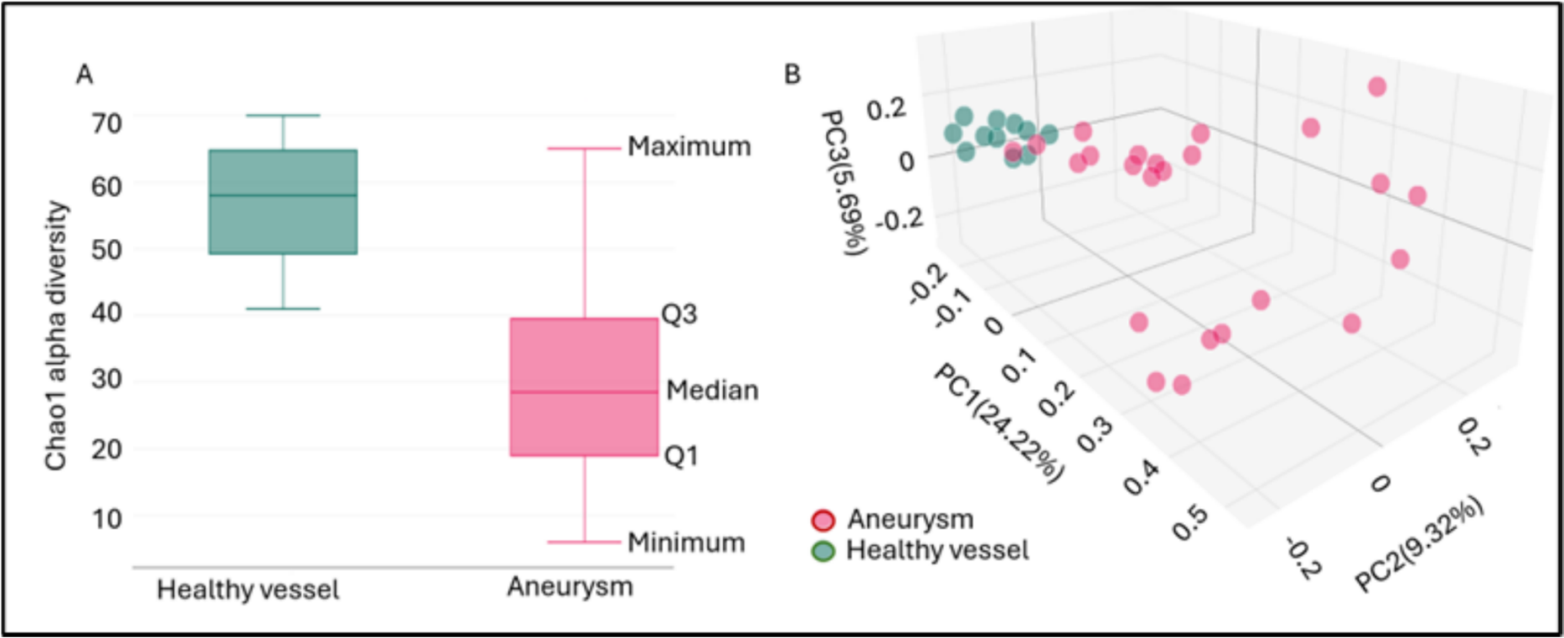
Mycobiome Chao1 alpha diversity (**A**) and Jaccard beta diversity PCoA (**B**) of healthy blood vessel wall and aneurysm wall samples: Boxplot displaying the data distribution with minimum, first quartile (Q1), median, third quartile (Q3), and maximum values, illustrating central tendency and variability, PC: principal coordinate

When examining the mycobiome composition of healthy and aneurysmal vessel walls, we found significant differences in both alpha diversity and beta diversity values. Figure 4a, shows that the Chao1 alpha diversity values of the aneurysmal vessel wall mycobiome is significantly lower than the value of the healthy vessel wall mycobiome (*p* ≤ 0.001). Figure 4b Jaccard beta diversity PCoA demonstrates that healthy vessel wall samples and aneurysm samples are arranged into significantly distinct groups (*p* = 0.001).

The genera *Tomentella* (20.94%) and *Didymosphaeria* (13.36%) are present with exceptionally high average abundance in the composition of the healthy vascular wall mycobiome, in contrast, the main participant in the aneurysmal mycobiome is the genus *Malassezia* (28.9%). There was also a demonstrable significant difference in the abundance of the *Venturiales* genus between the two groups in favor of AAA. Table 1 compares the average abundance and significance values of the most common genera constituting healthy and aneurysmal vessel wall mycobiomes.

**Table 1:** Median abundance and significance values of the most common fungal genera in healthy and aneurysmal vessel wall, listed in descending order from highest frequency in the given sample type

Genera	Healthy vessel wall (%)	Aneurysma wall (%)	*p* value
*Tomentella*	20.94	3.86	0.01
*Didymosphaeria*	13.36	4.07	0.09
*Malassezia*	1.92	28.9	0.01
*Venturiales*	2.09	6.37	0.01
*Penicillium*	3.55	5.00	0.44
*Aspergillus*	1.19	3.64	0.38
*Alternaria*	1.37	2.34	0.51

**Fig. 5:**
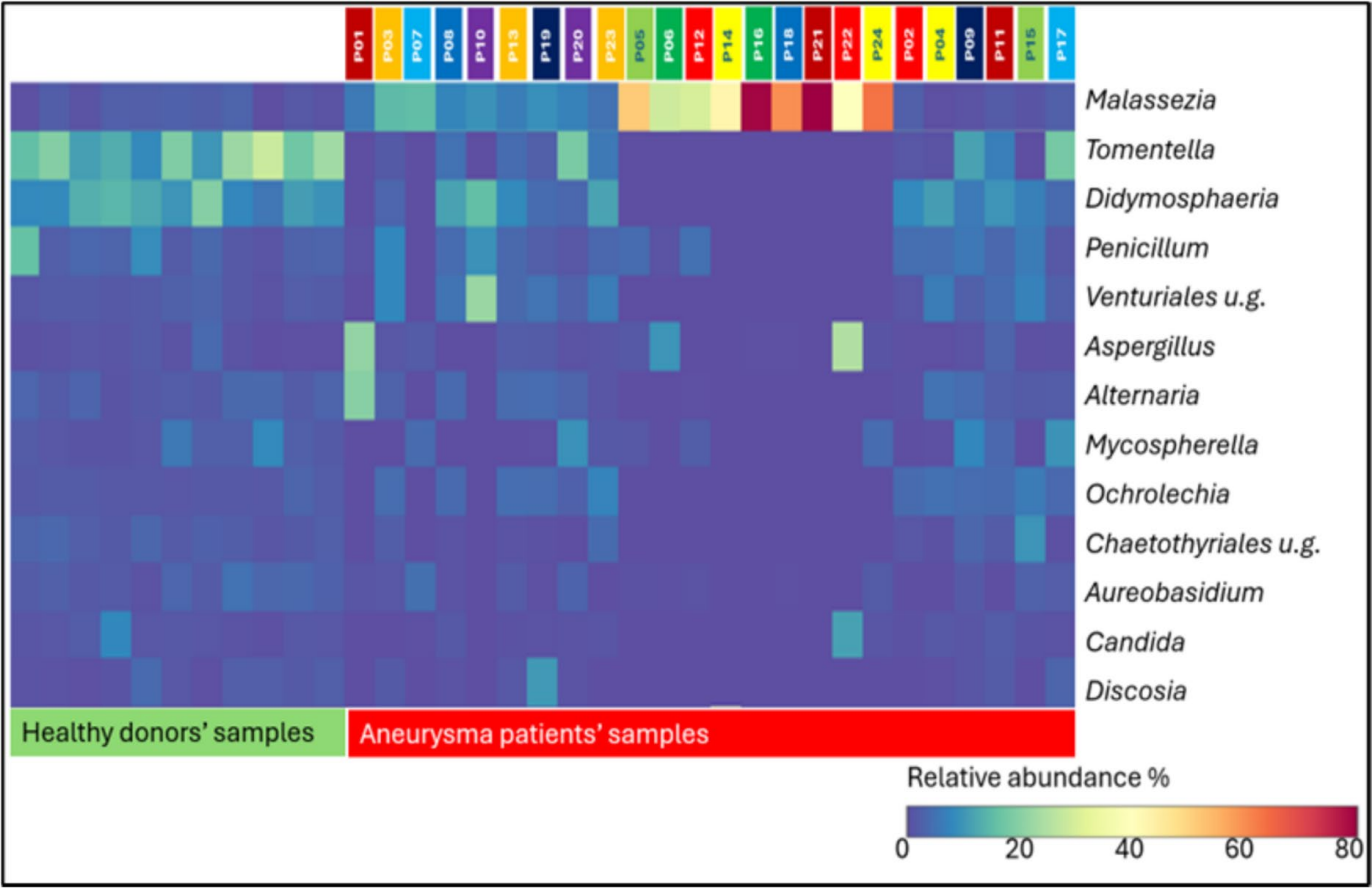
Heatmap representation of healthy and aortic aneurysm mycobiome composition

The mycobiome composition of aneurysm samples is organized into three distinct groups based on the amount of *Malassezia* (Fig. 5). In cases of high abundance of the *Malassezia* genus, *Tomentella* and *Didymosphaeria* are absent in the samples. As the abundance of *Malassezia* decreases, the abundance of the other two genera increases. However, even in the absence of *Malassezia* in the aneurysm mycobiome, *Tomentella* (7.8%) and *Didymosphaeria* (9.3%) appear in lower abundance compared to the healthy vessel wall (20.94% and 13.36%, respectively).

**Fig. 6:**
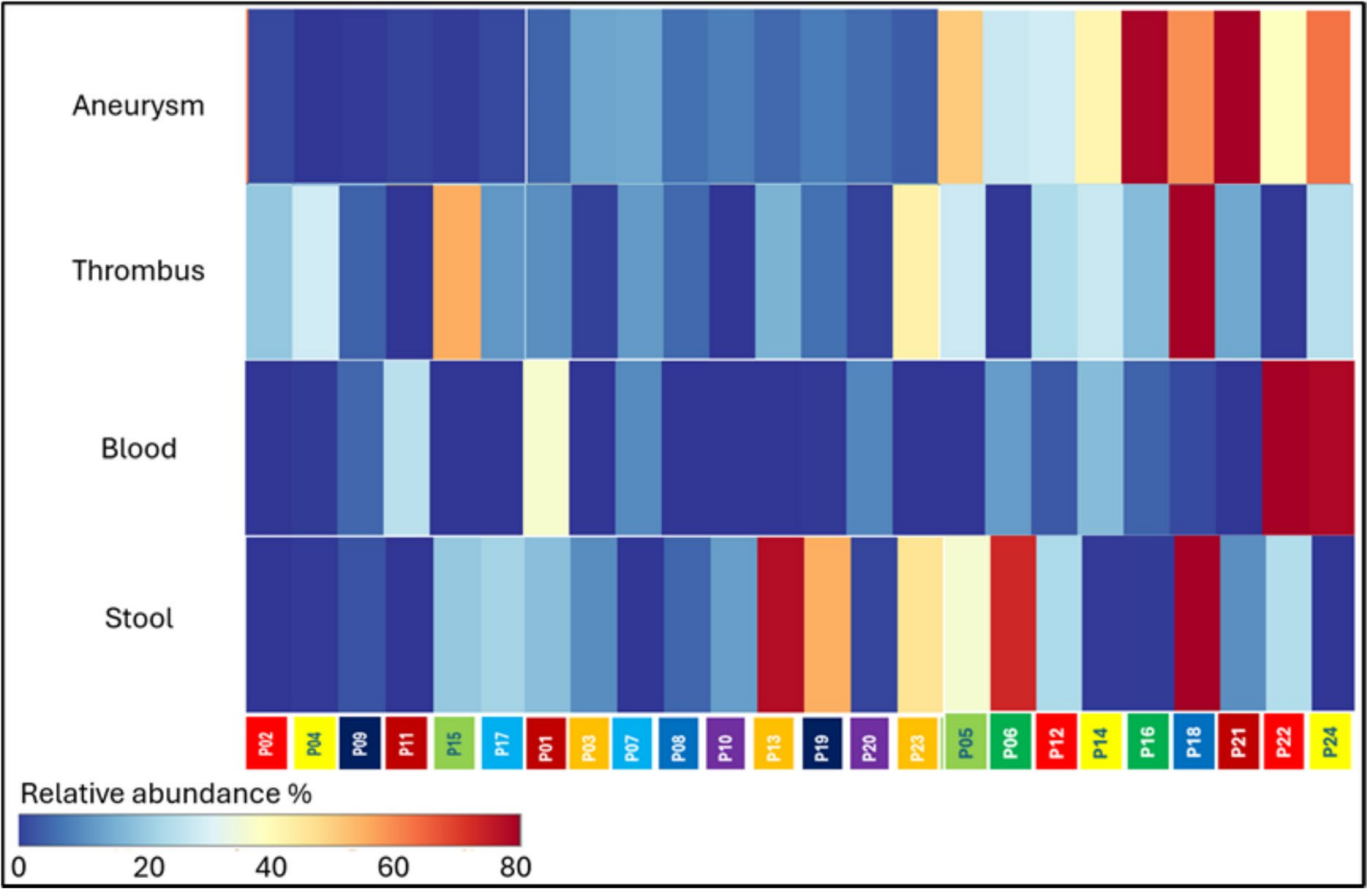
Heatmap about the *Malassezia* genus relative abundance in the aneurysm, thrombus, blood and stool in individual patients

*Malassezia* genus, which is barely detectable in the healthy vessel wall, was detected in the aneurysm mycobiome with an abundance of up to 80%. However, the abundance of the *Malassezia* genus, broken down into individuals, showed no correlation between the different sample types. Based on Fig. 6, a trend emerges that patients with vascular walls with high *Malassezia* abundance also more often have high abundance of the fungus in their stool, but exceptions exist. It cannot be proven that the *Malassezia* yeast found in the aneurysm was transferred from the stool to the aneurysm wall via the blood.

## Discussion

The mycobiome is now recognized as a critical part of the human microbial ecosystem, playing a significant role in both health and disease. Mycobiome research is currently in its infancy. First, it was necessary to determine the gene sequences of cultivated fungi and create data banks so that the results of next generation sequencing (NGS) metagenome studies could be associated with a correctly identified fungal name. Nowadays the most appropriate method is NGS-based amplicon sequencing targeting the ITS region to determine the fungal composition of the microbial community [21]. Accurately determining the structure of the mycobiome community is still a difficult bioinformatic task. Identification of fungal taxa within the mycobiome is currently successful at the genus level. Current scientific knowledge is still incomplete about the gene sequences of fungi found in the human body. The identification of the sequences per sample was therefore only partially successful, between 50 and 100%, and some sequences were unidentified or misidentified (as mushrooms) based on the most complete match with current databases.

The significant variation in alpha and beta diversity across our stool, blood, thrombus and vascular wall sample types underscores the compartmentalization of fungal communities within the human body. This finding aligns with prior research indicating that fungal populations differ markedly in gut, oral cavity, respiratory tract, skin or urinary tract due to unique local environments and selective pressures [22]. In the case of obesity, which is known to be a risk factor for AAA [15], and in studies of AAA patients, there is evidence of disease-related changes in the fecal mycobiome. Studies have shown that *Candida* species are often enriched in the gut microbiome of AAA patients, while the abundance of beneficial protective yeasts such as *S. cerevisiae* is reduced [18, 23]. The therapeutic potential of *S. cerevisiae* was demonstrated in animal models, where supplementation reduced aneurysm progression by improving gut barrier integrity and reducing systemic inflammation [18]. Some of our AAA patients had high *Candida* abundance in their stool samples, and only in 3 out of 24 patients were able to detect *Saccharomyces* in their stool samples. However, the stool mycobiome composition of our patients was characterized by the dominant genus *Malassezia* in most cases.

The mechanisms by which fungi influence AAA development remain speculative but could involve immune modulation, enzymatic activity, or interactions with bacterial populations. For instance, *Candida* species are known to promote inflammation through Th17 responses, potentially exacerbating vascular damage [24, 25]. Although most fungi do not produce butyrate on their own, they interact with bacteria in the gut, influencing the production of short-chain fatty acids (SCFAs) like butyrate. Beneficial fungi like *S. cerevisiae* can increase butyrate levels, which indirectly supports gut health and potentially reduces aneurysm progression [18]. Our findings, characterized by a high abundance of *Candida* and a low abundance of *Saccharomyces* in the fecal, support the hypothesis that fungal dysbiosis contributes to the pathogenesis of AAA through complex host-microbe interactions.

Following the principle that changes in fecal bacterial composition can partially be traced in the blood, thrombus and AAA vessel wall microbiome [12], we expected to detect components of the fecal mycobiome in the blood and vessel wall. The cohorts of sample types from all examined individuals, not significantly differentiated in fungal genus abundance, and no fungal taxon showed increased presence in any specific sample type. Individual analyses of gut, blood, thrombus and AAA vessel wall mycobiome compositions revealed conflicting abundances across personal samples. The translocation of fungal genera from feces through the bloodstream to the thrombus or AAA vessel wall could not be confirmed. Further studies are required to determine whether the mycobiome detected in the aneurysmal wall originates from the oral cavity, urinary tract, or skin.

The significant role of the human mycobiome in health and disease in the gut, oral cavity, urinary tract, or skin has been investigated by several studies [26], but the presence of the mycobiome in human blood or vascular wall as part of a normal mycobiome is not well documented or recognized. We used preserved DNA samples isolated from femoral artery of healthy organ donors from our previous research [19] to explore the composition of the healthy mycobiome of the vessel wall. Our study reveals significant differences in the mycobiome composition between healthy and aneurysmal vessel walls, as evidenced by variations in both alpha and beta diversity values. Notably, the genera *Tomentella* and *Didymosphaeria* were found to be highly abundant in the healthy vascular wall mycobiome, with average abundances of 20.94% and 13.36%, respectively. In contrast, the aneurysmal mycobiome was predominantly characterized by the genus *Malassezia*, which accounted for 28.9% of the composition. Additionally, the *Venturiales* genus was present in both healthy (2.09%) and AAA (6.37%) vessel walls with only a low median abundance, but the difference between the two groups was significant.

While the primary focus of *Venturiales* has been on plant pathogens, such as those in the genus *Venturia* which cause significant damage to fruit crops, there are also species within this order that can impact human health [27]. While rare, infections caused by melanized fungi members of *Venturiales*, can be serious. They can range from mild cutaneous disorders to systemic or disseminated infections, particularly in immuno-compromised individuals [28, 29].

The genus *Tomentella* is a part of the fungal kingdom, commonly found in soil and decaying organic matter. However, recent studies have also identified *Tomentella* in human microbiomes, including its presence in oral cavity, gut or brain tumor tissue [30–32]. Detailed studies on *Tomentella* in human health are limited, and its specific functions and interactions within the human body remain poorly understood. Research results to date on the beneficial or harmful effects of *Tomentella* are controversial, for example, their increased abundance in the fecal microbiome has been confirmed in rheumatoid arthritis and undifferentiated connective tissue diseases or in the blood and tumor tissue of brain tumor patients. In contrast, animal studies show that *Tomentella* is one of the few fungal genera that produce butyrate and acetate, short-chain fatty acids that are important for gut health and immune system regulation [33, 34].

*Didymosphaeria* is primarily known for its associations with plants, where it can act as a saprobe, endophyte, or pathogen. While there is a growing interest in the fungal diversity within human and animal microbiomes, specific infections by *Didymosphaeria* in mammals have not been reported. In mammalian mycobiome studies, elevated abundance of *Didymosphaeria* has been reported in bat gut microbiome and human brain tumors in scientific literature [32, 35].

The genus *Malassezia* is a prominent component of the human mycobiome, particularly on the skin, where it is the major fungal colonizer. It is present on nearly all humans and plays a complex role in skin health, sometimes contributing to conditions like atopic dermatitis due to sensitization to its antigens [36]. The *Malassezia* genus is known to be an important participant in both the oral mycobiome and the fecal mycobiome [30]. High intestinal abundance of *Malassezia* has been linked to several pathological conditions including inflammatory bowel disease, colorectal cancer or pancreatic cancer [37]. *Malassezia* species have been associated with gut inflammation and have been found in increased abundance in patients with AAA, suggesting that potential influence on systemic inflammation could indirectly affect vascular integrity [18]. There are no direct reports of *Malassezia* causing mycotic aneurysms, which are typically associated with fungal infections like *Aspergillus, Candida, Mucor* or *Penicillium* that directly invade vessel walls. The aforementioned infections are usually associated with systemic diseases and immunosuppressed conditions [38, 39]. In some of our AAA vessel wall samples, the genus *Malassezia* was present with an exceptionally high abundance, and its quantity increased in inverse proportion with the presence of the genera *Tomentella* and *Didymosphaeria*. We can neither confirm nor refute the existence of the gut-blood–vascular wall mycobiome axis and the translocation of *Malassezia* or *Tomentella* genera from the intestine to the vascular wall. It is possible that the primary colonization surface of these fungal taxa may be related to other anatomical areas of the human body, such as the nasal or oral cavity, urinary tract, genitals, or skin. However, our results of mycobiome abundance measured in pathological and healthy vascular walls agree with previous research that demonstrated the preventive properties of *Tomentella* against inflammation and the pro-inflammatory properties of *Malassezia*.

Limitations of the present study: The incomplete and incorrect identification of the obtained sequences was presumably due to the fact that the currently available databases are not yet suitable for identifying all fungal DNA associated with humans. The patient population most affected by abdominal aortic aneurysm predominantly consists of older males, whereas the arterial tissue samples used as healthy negative controls were obtained from healthy young donors eligible for multiorgan donation. Consequently, the study cohort lacked age diversity and differed significantly from the arterial wall microbiome healthy control group. Significant differences observed in the mycobiome composition may also be influenced by the differing age and health status of the two study groups. Unfortunately, obtaining age-matched healthy aortic tissue samples is extremely challenging due to ethical and practical constraints, as healthy aortic tissue is rarely available except from organ donors or autopsies, who often differ in age from patients with aneurysms. This limitation is common in aortic disease research and has been noted in previous studies as well [40, 41]. Suitable stool and blood samples for microbiome analysis from the arterial wall negative control individuals were not available. Furthermore, this study did not investigate the comorbidities, regular medication use, or other clinical parameters of the AAA patients. Examining these factors in a larger cohort could provide more precise and robust insights into the relationships between the disease and the mycobiome composition across different compartments.

## Conclusion

Our current study is the first to investigate not only the fecal but also the blood, thrombus, and aneurysm wall mycobiome in AAA patients. The four distinct anatomical locations exhibit differing mycobiome compositions in both alpha and beta diversity. Upon individual examination of the four sample types, we cannot confirm the existence of a gut-blood-vessel wall axis. The fungal genera detected in the vessel wall may have translocated from sites other than the gut. Comparing the mycobiome of healthy and aneurysmal vessel walls, we found significantly higher abundance of the genus *Tomentella* in healthy regions, while *Venturiales* and *Malassezia* were more abundant in aneurysms. Literature suggests that *Malassezia* is involved in pro-inflammatory processes, whereas *Tomentella* participates in anti-inflammatory processes in the gut. Our findings regarding the vessel wall mycobiome suggest a potential association between the presence of *Tomentella* and *Malassezia* and the progression of AAA. These observations should be interpreted as preliminary and hypothesis-generating, warranting further investigation in studies specifically designed to assess causal relationships. Our results underscore the significance of fungi in the progression of AAA and propose potential therapeutic pathways, including the use of targeted antifungal adjunct therapies to reduce inflammation and prevent aneurysm formation.
